# Albuterol-Budesonide Pressurized Metered Dose Inhaler in Patients With Mild-to-Moderate Asthma: Results of the DENALI Double-Blind Randomized Controlled Trial

**DOI:** 10.1016/j.chest.2023.03.035

**Published:** 2023-03-30

**Authors:** Bradley E. Chipps, Elliot Israel, Richard Beasley, Reynold A. Panettieri, Frank C. Albers, Robert Rees, Lynn Dunsire, Anna Danilewicz, Eva Johnsson, Christy Cappelletti, Alberto Papi

**Affiliations:** a Capital Allergy and Respiratory Disease Center, Sacramento, CA; b Brigham and Women’s Hospital, Harvard Medical School, Boston, MA; c Medical Research Institute of New Zealand, Wellington, New Zealand; d Rutgers Institute for Translational Medicine and Science, Child Health Institute of New Jersey, Rutgers, State University of New Jersey, New Brunswick, NJ; e Avillion US Inc., Northbrook, IL; f Avillion LLP, London, UK; g BioPharmaceuticals R&D, AstraZeneca, Cambridge, UK; h BioPharmaceuticals R&D, AstraZeneca, Gothenburg, Sweden; i BioPharmaceuticals R&D, AstraZeneca, Durham, NC; j Department of Translational Medicine, University of Ferrara, Ferrara, Italy

**Keywords:** albuterol-budesonide, albuterol-ICS, asthma, bronchodilators, inflammation, inhaled corticosteroid, rescue therapy, short-acting β_2_-agonist

## Abstract

**Background:**

In the phase 3 MANDALA trial, as-needed albuterol-budesonide pressurized metered-dose inhaler significantly reduced severe exacerbation risk vs as-needed albuterol in patients with moderate-to-severe asthma receiving inhaled corticosteroid-containing maintenance therapy. This study (DENALI) was conducted to address the US Food and Drug Administration combination rule, which requires a combination product to demonstrate that each component contributes to its efficacy.

**Research Question:**

Do both albuterol and budesonide contribute to the efficacy of the albuterol-budesonide combination pressurized metered-dose inhaler in patients with asthma?

**Study Design and Methods:**

This phase 3 double-blind trial randomized patients aged ≥ 12 years with mild-to-moderate asthma 1:1:1:1:1 to four-times-daily albuterol-budesonide 180/160 μg or 180/80 μg, albuterol 180 μg, budesonide 160 μg, or placebo for 12 weeks. Dual-primary efficacy end points included change from baseline in FEV_1_ area under the curve from 0 to 6 h (FEV_1_ AUC_0-6h_) over 12 weeks (assessing albuterol effect) and trough FEV_1_ at week 12 (assessing budesonide effect).

**Results:**

Of 1,001 patients randomized, 989 were ≥ 12 years old and evaluable for efficacy. Change from baseline in FEV_1_ AUC_0-6h_ over 12 weeks was greater with albuterol-budesonide 180/160 μg vs budesonide 160 μg (least-squares mean [LSM] difference, 80.7 [95% CI, 28.4-132.9] mL; *P* = .003). Change in trough FEV_1_ at week 12 was greater with albuterol-budesonide 180/160 and 180/80 μg vs albuterol 180 μg (LSM difference, 132.8 [95% CI, 63.6-201.9] mL and 120.8 [95% CI, 51.5-190.1] mL, respectively; both *P* < .001). Day 1 time to onset and duration of bronchodilation with albuterol-budesonide were similar to those with albuterol. The albuterol-budesonide adverse event profile was similar to that of the monocomponents.

**Interpretation:**

Both monocomponents contributed to albuterol-budesonide lung function efficacy. Albuterol-budesonide was well tolerated, even at regular, relatively high daily doses for 12 weeks, with no new safety findings, supporting its use as a novel rescue therapy.

**Clinical Trial Registration:**

ClinicalTrials.gov; No.: NCT03847896; URL: www.clinicaltrials.gov

## Graphical Abstract

**Figure:**
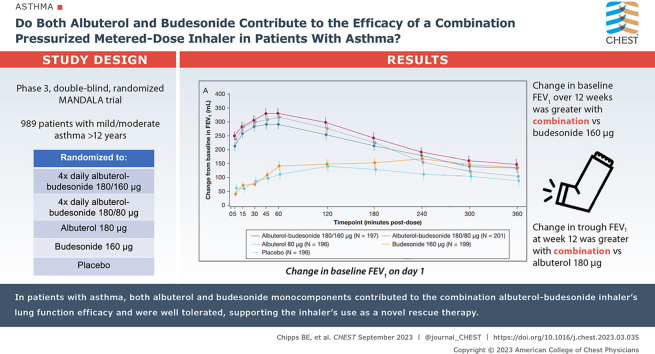

**Take-home Points**

**Study Question:** Do both albuterol and budesonide contribute to the efficacy of albuterol-budesonide pressurized metered dose inhaler (pMDI)?

**Results:** Albuterol and budesonide both contributed to the lung function efficacy of the albuterol-budesonide combination, as assessed by change from baseline in FEV_1_ area under the curve from 0 to 6 h over 12 weeks and in trough FEV_1_ at week 12, with no new safety findings observed even with regular use of relatively high doses over 12 weeks.

**Interpretation:** This trial confirms the contribution of both monocomponents to the lung function efficacy of albuterol-budesonide combination pMDI.

Short-acting β_2_-agonist (SABA) rescue inhalers provide quick relief of asthma symptoms, but do not treat underlying airway inflammation.^1^ Their use is highly prevalent among patients with asthma^2^; however, increasing exposure to SABA is associated with an increased severe exacerbation risk,^2^^,^^3^ as well as increases in asthma-related ED visits or hospital admissions^2^ and asthma-related mortality.^4^^,^^5^

In view of this and the proven benefit of adding inhaled corticosteroids (ICS), the Global Initiative for Asthma (GINA) recommends that SABA not be used alone without ICS therapy.^1^ Furthermore, GINA advocates a combination inhaler containing both the fast-acting bronchodilator formoterol and an ICS as preferred rescue treatment strategy for all disease severities in patients aged ≥ 12 years, based on the evidence of greater efficacy in reducing severe exacerbation risk compared with SABA rescue therapy.^1^ A SABA-ICS combination inhaler is now included as an alternative rescue option for patients ≥ 12 years old.^1^ The US National Asthma Education and Prevention Program includes as-needed concomitant SABA and ICS as a preferred treatment strategy in patients aged ≥ 12 years with mild persistent asthma (step 2), and daily and as-needed ICS-formoterol for patients with moderate-to-severe persistent asthma (steps 3 and 4).^6^ While both GINA and the US National Asthma Education and Prevention Program include ICS-formoterol in their recommendations for maintenance therapy and as rescue for many patients, no ICS-formoterol product is approved by the US Food and Drug Administration (FDA) for as-needed use.

A novel combination of albuterol and budesonide^7^^,^^8^ in a single pressurized metered dose inhaler (pMDI) was developed using Co-Suspension technology as an albuterol-ICS rescue therapy that could be used on top of any ICS-containing maintenance therapy, and in January 2023 became the first asthma rescue inhaler to be approved by the FDA for as-needed use to reduce the risk of exacerbations. Approval relied on the results from the MANDALA and DENALI phase III pivotal trials. In MANDALA, as-needed albuterol-budesonide 180/160 μg pMDI significantly reduced the risk of severe exacerbations by 27% compared with as-needed use of albuterol alone among patients aged ≥ 12 years with uncontrolled moderate-to-severe asthma receiving a wide range of ICS-containing maintenance therapies.^9^

DENALI was conducted to fulfill the requirements of the FDA combination rule (21 CFR 300.50), which requires demonstration that each component of a combination product contributes to its efficacy. The objective of DENALI was to demonstrate the contribution of each monocomponent to the efficacy of albuterol-budesonide pMDI in patients with mild-to-moderate asthma. This required a study design incorporating scheduled dosing (despite the product being intended and approved for as-needed use). The hypothesis was that each of the monocomponents in the combination individually contributes to lung function efficacy.

## Study Design and Methods

### Trial Design and Patients

DENALI was a phase 3, randomized, double-blind, multicenter trial^10^ conducted at 126 sites in the United States, Europe, and South America between March 2019 (first patient enrolled) and July 2021 (last patient completed). The trial comprised a 2- to 4-week run-in period, a 12-week randomized treatment period, and a 2-week safety follow-up (e-Fig 1).

The trial was performed in accordance with the Declaration of Helsinki and the International Council for Harmonisation Good Clinical Practice guidelines. The protocol was approved by relevant independent ethics committees or institutional review boards, and all patients (or their guardian if aged < 18 years) provided written informed consent.

Patients aged ≥ 4 years with mild-to-moderate asthma (GINA 2018 criteria)^11^ receiving as-needed SABA or low-dose maintenance ICS plus as-needed SABA therapy at a stable dose for ≥ 30 days prior were included. Additional entry criteria included prebronchodilator FEV_1_ ≥ 50% to < 85% predicted normal value (≥ 50% for patients aged 4-17 years) and reversibility of FEV_1_ (≥ 15% increase vs baseline). Asthma control was not specified as an entry criterion. Main exclusion criteria were the presence of COPD or other significant lung disease, any systemic corticosteroid (SCS) use in the 3 months prior or chronic SCS use (≥ 3 weeks) in the preceding 6 months, or asthma-related hospitalization within 6 months before screening.

### Treatment

On entering the run-in period, patients discontinued preenrollment SABA and any low-dose ICS, and received single-blind placebo qid and sponsor-provided albuterol (Ventolin; GlaxoSmithKline) used as needed for symptoms during run-in; only the latter was continued throughout the randomized treatment period. Patients aged ≥ 12 years were then randomized by a centralized Interactive Web Response System 1:1:1:1:1 to qid albuterol-budesonide 180/160 μg, albuterol-budesonide 180/80 μg, budesonide 160 μg, albuterol 180 μg, or placebo via pMDI for 12 weeks. Each dose was administered as two actuations of albuterol-budesonide 90/80 μg or 90/40 μg, budesonide 80 μg, albuterol 90 μg, or placebo. Children aged 4 to 11 years were randomized 1:1:1 to qid albuterol-budesonide 180/80 μg, albuterol 180 μg, or placebo.

Randomization for patients aged ≥ 12 years was stratified by pretrial background therapy (ICS vs non-ICS), Asthma Control Questionnaire-7 (ACQ-7) score (≤ 1.5 vs > 1.5), and age (≥ 12-17 vs ≥ 18 years). Patients and trial investigators were blinded to treatment allocation. The different kit types corresponding to each of the treatment groups were visually identical, protecting the blind through their similarity in appearance.

Patients were instructed to take trial medication on waking and to distribute doses equally throughout the day, taking the final dose before going to sleep. Additional asthma medications (other than sponsor-provided albuterol for as-needed use) and spacer devices were not permitted.

### Trial Procedures, Assessments, and End Points

In-clinic visits were at weeks 1, 4, 8, and 12; the full scheduling of visits and assessments is shown in e-Figure 1. Spirometry assessments were completed at the trial site 60 and 30 min predose and 5, 15, 30, 45, 60, 120, 180, 240, 300, and 360 min postdose of investigational product; ACQ-7 was also assessed (among patients with a baseline score ≥ 1.5) at these visits. Albuterol rescue use was not permitted within 6 h of spirometry assessments. All patients were provided an eDiary to record peak expiratory flow measurements, trial medication use, and asthma symptoms.

The dual-primary efficacy end points determined the therapeutic contribution of each monocomponent to lung function efficacy; these were (1) change from baseline in FEV_1_ area under the curve from 0 to 6 h (AUC_0-6h_) averaged over the 12-week randomized treatment period to assess the contribution of albuterol, and (2) change from baseline in trough FEV_1_ at week 12 to assess the contribution of budesonide.

Secondary efficacy end points included time to onset of FEV_1_ response (defined as a ≥ 15% improvement in pretreatment FEV_1_ within 30 min of the first inhalation of trial medication) and duration of response on day 1; postdose ACQ-7 responders (response defined as a ≥ 0.5-point reduction from baseline^12^, ^13^, ^14^) at week 12 among patients with a baseline score ≥ 1.5; and trough FEV_1_ at week 1. Scoring on the ACQ-7 ranges from 0 to 6, with lower scores indicating better asthma control (minimum clinically important difference, −0.5 point).

Severe exacerbations were collected as an exploratory end point (defined as at least one of the following: a ≥ 3-day course of oral corticosteroids or corresponding single injectable dose, an ED or urgent care visit due to asthma that required SCS, or hospitalization due to asthma). Treatment adherence was calculated from eDiary records as the number of inhalations of trial medication taken as a proportion of the expected 8 inhalations/d of trial medication during the randomized treatment period.

Safety assessments included adverse events (AEs) and serious AEs, and monitoring vital signs, clinical chemistry and hematology parameters, and ECG recordings during the randomized treatment period.

### Statistical Analysis

A sample size of 1,000 patients was calculated to provide 85% power to detect a true treatment difference of 100 mL in change from baseline in trough FEV_1_ at week 12 and assuming an SD of 320 mL, for the relevant treatment comparisons in the hierarchical testing procedure. This final sample size was the result of two blinded sample size reestimations (see the online supplement).

All patients aged ≥ 12 years receiving any amount of trial medication, and who had at least one efficacy assessment, were included in the efficacy analyses classified by the trial medication to which they were randomized. Children aged 4 to 11 years were excluded, a priori, from the primary efficacy analyses because of the low numbers enrolled. All patients (regardless of age) receiving any trial medication were evaluated for safety, classified by the treatment they received.

Change from baseline in FEV_1_ AUC_0-6h_ was calculated using the trapezoidal rule, normalized by dividing by the time (in hours) from dosing to the last measurement included. Change from baseline in trough FEV_1_ was calculated as the average of the 60- and 30-min predose spirometry values before dosing. Baseline FEV_1_ for both end points was defined as the predose value collected at the randomization visit (day 1). In patients with only one predose assessment for trough FEV_1_, the value was calculated from the single measurement. The dual-primary end points were analyzed using a repeated-measures linear model, with baseline FEV_1_, percentage bronchodilator responsiveness to albuterol, and age included as continuous covariates, and visit, treatment, treatment-by-visit interaction, and pretrial background therapy (ICS vs non-ICS) as categorical covariates.

**Figure 1:**
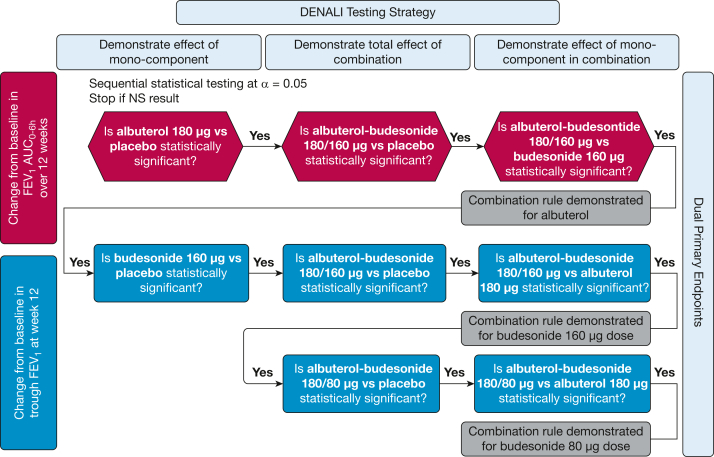
Sequential testing of dual-primary end points. For FEV_1_ AUC_0-6h_ over 12 weeks (which evaluated the contribution of albuterol 180 μg), albuterol-budesonide 180/80 μg was not selected for testing in the statistical hierarchy, as the albuterol dose is the same in both albuterol-budesonide 180/160 μg and 180/80 μg. FEV_1_ AUC_0-6h_ = FEV_1_ area under the curve from 0 to 6 h; NS = nonsignificant.

The primary efficacy analyses (which included patients aged ≥ 12 years) assumed continuation of randomized treatment for the duration of the trial, regardless of actual adherence. Missing data were assumed to be missing at random. For the dual-primary end points, the overall type I error for the preplanned treatment comparisons was controlled by testing sequentially through an eight-step hierarchical testing procedure in a prespecified order (Fig 1). Testing proceeded to the next comparison only if the present comparison was statistically significant at the 5% significance level (α = 0.05, two-sided). For FEV_1_ AUC_0-6h_ over 12 weeks (which evaluated the contribution of albuterol 180 μg), the comparisons of albuterol-budesonide 180/80 μg vs placebo and vs budesonide were not included in the testing hierarchy as the contribution of albuterol to the combination was captured in the first three testing steps with albuterol-budesonide 180/160 μg. Analysis of the dual-primary efficacy end points including patients of all ages (ie, ≥ 4 years) was also planned as a supportive analysis. Sensitivity analyses (tipping point analyses and reference-based imputation analyses), performed to assess the robustness of the primary efficacy analysis to alternative assumptions about missing data, are detailed in the online supplement.

Analyses of the secondary efficacy end points were not type I error rate controlled, and therefore *P* values are nominal. The number and percentage of FEV_1_ responders on day 1 was calculated. Median time to onset (minutes) of the FEV_1_ response was compared among FEV_1_ responders in each treatment group, and CIs for the median treatment difference were calculated using the Hodges-Lehmann method. Descriptive statistics for duration of response (minutes) were reported by treatment group. ACQ-7 response was compared between groups using a logistic regression model that was adjusted for baseline values (pretrial background ICS, ACQ-7 score, postdose FEV_1_ % predicted normal, and age).

In addition to the statistical analyses described above, all end points were summarized by treatment group when appropriate. All tests were two-sided and at a 5% level of significance, unless otherwise stated.

## Results

### Patients

**Figure 2:**
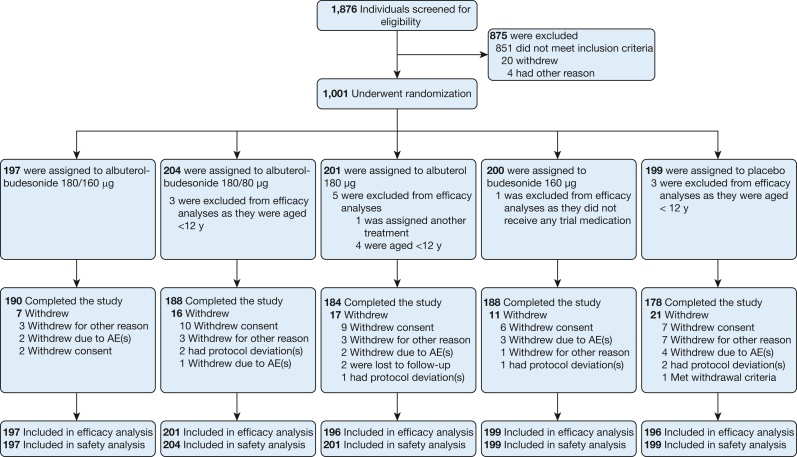
Patient disposition. The efficacy analysis included patients aged ≥ 12 y, and the safety analysis included patients of all ages. AE = adverse event.

Of the 1,001 patients randomized, 1,000 (10 children aged 4-11 years and 990 patients ≥ 12 years) were assessed for safety (one patient did not receive any trial medication) and 989 patients aged ≥ 12 years were included in the primary efficacy analyses (one duplicate patient was excluded) (Fig 2). In total, 928 patients (92.7%) completed the trial. There were no major disruptions to trial conduct because of COVID-19, and only seven patients (0.7%) missed any trial visits as a result. Patient demographics and clinical characteristics at baseline are presented in Table 1 and e-Table 1. At baseline, patients had a mean ACQ-7 score of 2.2 (median, 2.1), indicating poor asthma control.

**Table 1:** Baseline Demographic and Clinical Characteristics: Efficacy Population, ≥ 12 Years

Characteristic	Albuterol-Budesonide 180/160 μg (n = 197)	Albuterol-Budesonide 180/80 μg (n = 201)	Albuterol 180 μg (n = 196)	Budesonide 160 μg (n = 199)	Placebo (n = 196)
Age, mean ± SD, y	50.0 ± 15.8	49.2 ± 16.2	47.8 ± 16.1	48.3 ± 15.8	49.2 (15.1)
Age group, No. (%)					
≥ 12 to < 18 y	4 (2.0)	7 (3.5)	5 (2.6)	5 (2.5)	4 (2.0)
≥ 18 to < 65 y	154 (78.2)	155 (77.1)	158 (80.6)	161 (80.9)	161 (82.1)
≥ 65 y	39 (19.8)	39 (19.4)	33 (16.8)	33 (16.6)	31 (15.8)
Female sex, No. (%)	125 (63.5)	127 (63.2)	119 (60.7)	120 (60.3)	124 (63.3)
Race, No. (%)					
White	179 (90.9)	185 (92.0)	164 (83.7)	180 (90.5)	172 (87.8)
Black or African American	14 (7.1)	12 (6.0)	29 (14.8)	18 (9.0)	18 (9.2)
Asian	1 (0.5)	0	0	0	1 (0.5)
American Indian or Alaska Native	1 (0.5)	0	0	0	1 (0.5)
Other	2 (1.0)	4 (2.0)	3 (1.5)	1 (0.5)	4 (2.0)
Prebronchodilator FEV_1_, mean ± SD					
Volume, L	2.1 ± 0.6	2.1 ± 0.7	2.2 ± 0.7	2.1 ± 0.7	2.1 ± 0.7
% predicted normal	68.8 ± 13.1	70.0 ± 14.6	69.5 ± 12.9	68.9 ± 13.8	68.3 ± 14.5
Reversibility in FEV_1_, mean ± SD, %^a^	28.2 ± 13.5	30.2 ± 12.9	27.4 ± 13.5	28.7 ± 14.4	28.8 ± 14.3
Pretrial background ICS therapy, No. (%)	93 (47.2)	97 (48.3)	94 (48.0)	95 (47.7)	94 (48.0)
ACQ-7 overall score, mean ± SD	2.2 ± 0.7	2.2 ± 0.6	2.2 ± 0.7	2.1 ± 0.6	2.2 ± 0.7

Data are for patients aged ≥ 12 y included in the efficacy analyses. ACQ-7 = Asthma Control Questionnaire (7 item); ICS = inhaled corticosteroid.

a Reversibility was tested during screening and calculated as follows: [postbronchodilator FEV_1_ (L) − prebronchodilator FEV_1_ (L)]/prebronchodilator FEV_1_ (L) × 100; data are missing for one subject in the albuterol 180 μg group. Screening was defined as the time before the first administration of sponsor-provided albuterol during the run-in period.

### Adherence to Trial Medication

During the randomized treatment period, patients recorded administering a median of 93.8% of their trial medication (mean ± SD, 89.4% ± 12.2%). Compliance was similar across treatment groups (e-Table 2).

### Efficacy

All efficacy data are presented for the primary efficacy analyses (patients aged ≥ 12 years) unless otherwise stated.

The prespecified treatment comparisons for the dual-primary efficacy end points are presented in Table 2. Change from baseline in FEV_1_ AUC_0-6h_ averaged over 12 weeks was significantly greater with albuterol-budesonide 180/160 μg than budesonide 160 μg (least-squares mean [LSM] difference, 80.7 mL; 95% CI, 28.4-132.9 mL; *P* = .003) and placebo (LSM difference, 161.9 mL; 95% CI, 109.4-214.5 mL; *P* < .001) (Table 2, e-Tables 3, 4).

**Table 2:** Dual-Primary Efficacy End Points: Efficacy Population, ≥ 12 Years

Primary Efficacy End Point	Step in Testing Sequence	Comparisons	LSM	Comparison Between Groups	
				Difference in LSM (95% CI)	*P* Value (Two-Sided)
FEV_1_ AUC_0-6h_, change from baseline over 12 wk, mL^a^	1	Albuterol 180 μg (n = 195) vs placebo (n = 196)	157.2 vs 96.7	60.5 (7.7-113.4)	.025
	2	Albuterol-budesonide 180/160 μg (n = 197) vs placebo (n = 196)	258.6 vs 96.7	161.9 (109.4-214.5)	< .001
	3	Albuterol-budesonide 180/160 μg (n = 197) vs budesonide 160 μg (n = 199)	258.6 vs 178.0	80.7 (28.4-132.9)	.003
Trough FEV_1_, change from baseline at week 12, mL	4	Budesonide 160 μg (n = 187) vs placebo (n = 175)	108.9 vs 35.6	73.3 (4.4-142.2)	.037
	5	Albuterol-budesonide 180/160 μg (n = 186) vs placebo (n = 175)	135.5 vs 35.6	99.9 (30.9-168.8)	.005
	6	Albuterol-budesonide 180/160 μg (n = 186) vs albuterol 180 μg (n = 172)	135.5 vs 2.7	132.8 (63.6-201.9)	< .001
	7	Albuterol-budesonide 180/80 μg (n = 184) vs placebo (n = 175)	123.5 vs 35.6	87.9 (18.8-156.9)	.013
	8	Albuterol-budesonide 180/80 μg (n = 184) vs albuterol 180 μg (n = 172)	123.5 vs 2.7	120.8 (51.5-190.1)	< .001

AUC_0-6h_ = area under the curve from 0 to 6 h postdose; LSM = least-squares mean.

a The LSM was calculated as the difference between treatment groups average over all time points during the randomized treatment period.

Change from baseline in trough FEV_1_ at week 12 was significantly greater with albuterol-budesonide 180/160 μg and 180/80 μg than albuterol 180 μg (LSM difference, 132.8 mL; 95% CI, 63.6-201.9 mL; and 120.8 mL; 95% CI, 51.5-190.1 mL, respectively; both *P* < .001) and placebo (LSM difference, 99.9 mL; 95% CI, 30.9-168.8 mL; *P* = .005, and 87.9 mL; 95% CI, 18.8-156.9 mL; *P* = .013, respectively) (Table 2, e-Table 3).

Tipping point analyses and reference-based imputation analyses were supportive of the primary analyses under conservative assumptions for missing data (e-Tables 5, 6).

Similar results were observed in the analysis of the dual-primary efficacy end points that included patients of all ages (e-Table 7).

**Figure 3:**
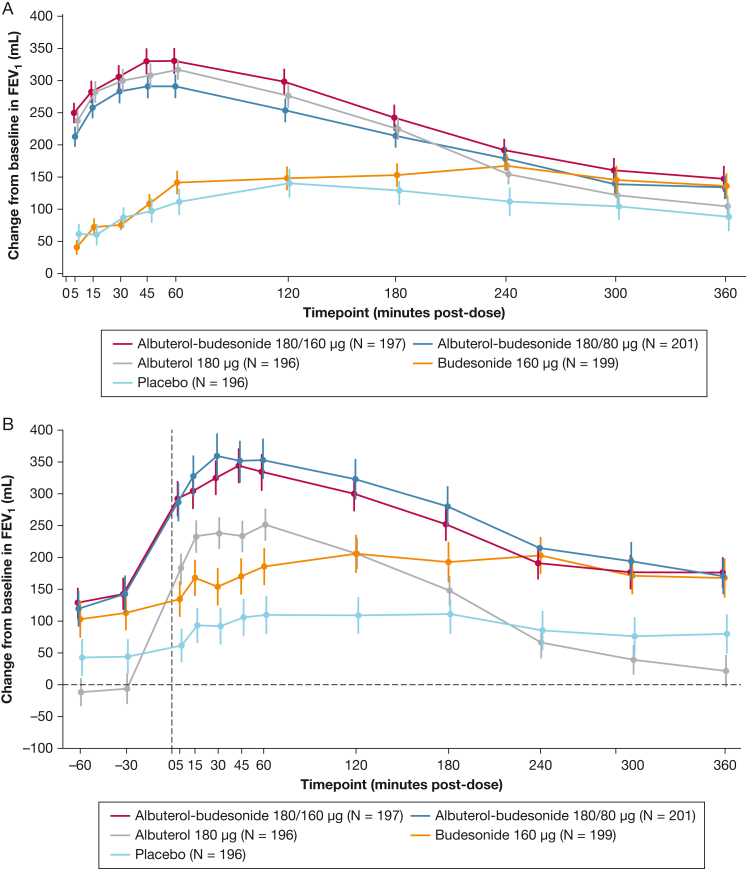
A and B, Change from baseline in FEV_1_ on day 1 (A) and week 12 (B) (serial spirometry profile).

The percentage of patients with an FEV_1_ response within 30 min on day 1 is shown in e-Figure 2. The median time to onset of FEV_1_ response was 7.5, 7.0, and 9.5 min with albuterol-budesonide 180/160 μg, albuterol-budesonide 180/80 μg, and albuterol, respectively, and the mean ± SD duration of response was 186.9 (122.5), 191.4 (127.3), and 168.2 (128.0) min, respectively. Figures 3A and B show serial spirometry profiles for the mean change from baseline in FEV_1_ on day 1 and week 12, respectively.

**Figure 4:**
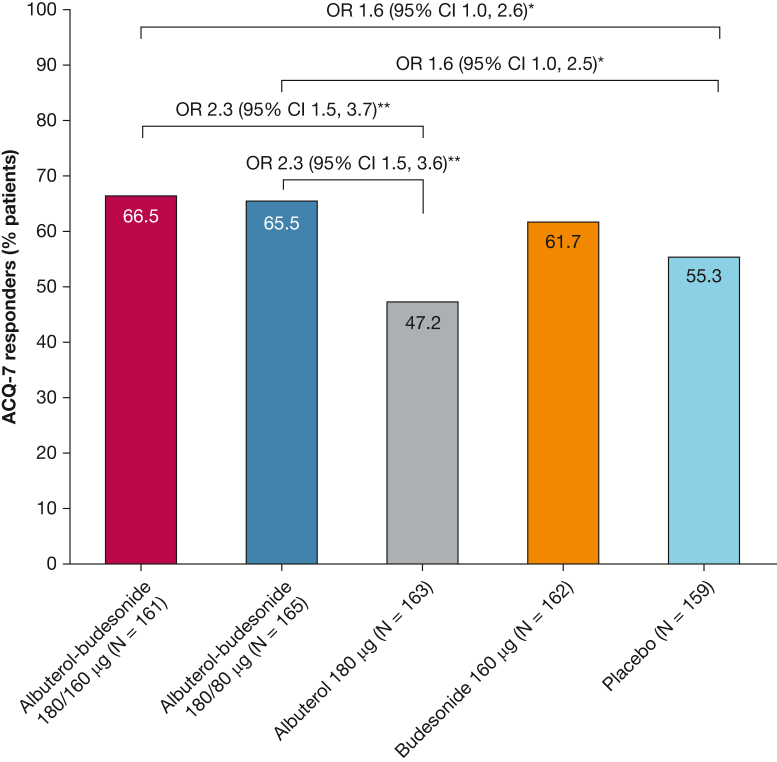
ACQ-7 response at week 12. Response defined as a decrease of ≥ 0.5 units from baseline at week 12 among patients with a baseline score ≥ 1.5. OR (95% CI) albuterol vs placebo: 0.7 (0.4, 1.1); *P* = .12. OR (95% CI) budesonide vs placebo: 1.4 (0.9, 2.2); *P* = .16. ∗*P* < .05 vs placebo; ∗∗*P* < .001 vs albuterol. ACQ-7 = Asthma Control Questionnaire-7.

Among patients with an ACQ-7 score > 1.5 at baseline, the odds of achieving an ACQ-7 response at week 12 were numerically higher with albuterol-budesonide 180/160 μg and 180/80 μg than with albuterol (OR, 2.3 [95% CI, 1.5-3.7] and 2.3 [95% CI, 1.5-3.6], respectively) (Fig 4).

The change from baseline in trough FEV_1_ at week 1 showed numerical improvement for albuterol-budesonide 180/160 μg and 180/80 μg vs albuterol (LSM difference, 107.9 mL [95% CI, 48.1-167.8 mL]; *P* < .001, and 72.8 mL [95% CI, 13.1-132.5 mL]; *P* = .017) (e-Table 8).

Over the 12-week randomized treatment period, severe exacerbations were experienced by fewer patients receiving albuterol-budesonide 180/160 μg, albuterol-budesonide 180/80 μg, or budesonide than those receiving albuterol or placebo (2.0%, 2.5%, 2.0%, 10.2%, and 7.1%, respectively; e-Table 9). Patients receiving albuterol-budesonide 180/160 μg and 180/80 μg reported using a mean of 1.3 and 1.4 rescue inhalations/d during the randomized treatment period compared with 1.9, 1.4, and 1.9 rescue inhalations/d in patients receiving albuterol, budesonide, and placebo, respectively (e-Table 10).

### Adverse Events

AEs were reported by 31% to 35% of patients across the treatment groups, with the most commonly reported being nasopharyngitis and headache (Table 3). A total of 15 serious AEs occurred in 13 patients, with one considered treatment related (an asthma exacerbation in the albuterol-budesonide 180/80 μg group). There were no deaths in the trial, and < 2% of patients discontinued the trial because of an AE (Table 3). Local ICS-associated AEs were reported in ≤ 2% of patients in the budesonide-containing arms (e-Table 11), and included dysphonia (2.0%, 0.5%, and 1.0% in the albuterol-budesonide 180/160 μg, albuterol-budesonide 180/80 μg, and budesonide groups, respectively), oral candidiasis (0.5%, 0.5%, and 0%, respectively), and oropharyngeal candidiasis (1.0%, 0%, and 0%, respectively).

**Table 3:** Adverse Events: Safety Population, All Ages

No. (%)	Albuterol-Budesonide 180/160 μg (n = 197)	Albuterol-Budesonide 180/80 μg (n = 204)	Albuterol 180 μg (n = 201)	Budesonide 160 μg (n = 199)	Placebo (n = 199)
Any AE	66 (33.5)	72 (35.3)	62 (30.8)	67 (33.7)	69 (34.7)
Any treatment-related AE	10 (5.1)	6 (2.9)	2 (1.0)	7 (3.5)	3 (1.5)
Any SAE	2 (1.0)	4 (2.0)	1 (0.5)	3 (1.5)	3 (1.5)
Any AE leading to treatment discontinuation	2 (1.0)	1 (0.5)	2 (1.0)	3 (1.5)	4 (2.0)
Deaths	0	0	0	0	0
AEs occurring in ≥ 2% of patients in any group^a^					
Nasopharyngitis	15 (7.6)	13 (6.4)	9 (4.5)	10 (5.0)	11 (5.5)
Headache	10 (5.1)	10 (4.9)	11 (5.5)	7 (3.5)	14 (7.0)
Diarrhea	2 (1.0)	2 (1.0)	4 (2.0)	2 (1.0)	4 (2.0)
Nausea	1 (0.5)	2 (1.0)	0	5 (2.5)	5 (2.5)
Upper RTI	2 (1.0)	3 (1.5)	1 (0.5)	4 (2.0)	2 (1.0)
Asthma	0	3 (1.5)	3 (1.5)	0	5 (2.5)
Oropharyngeal pain	2 (1.0)	2 (1.0)	2 (1.0)	5 (2.5)	0
Hypertension	4 (2.0)	2 (1.0)	2 (1.0)	0	2 (1.0)
COVID-19	2 (1.0)	1 (0.5)	0	2 (1.0)	4 (2.0)
Dysphonia	4 (2.0)	1 (0.5)	0	2 (1.0)	0

AE = adverse event; RTI = respiratory tract infection; SAE = serious adverse event.

a Preferred terms from the Medical Dictionary for Regulatory Activities (MedDRA) version 24.0 were used. Patients with multiple events in the same preferred term were counted only once in that preferred term.

## Discussion

Albuterol-budesonide pMDI is being developed as an asthma rescue therapy for as-needed use, combining a SABA for alleviation of symptoms with an ICS to simultaneously treat the accompanying increasing inflammation that precedes an exacerbation. The DENALI trial met both dual-primary end points, demonstrating the contribution of both monocomponents to the lung function efficacy of albuterol-budesonide pMDI. In addition, albuterol-budesonide demonstrated a similar rapid time to onset and duration of bronchodilation on day 1 to that of albuterol. Improved trough FEV_1_ with albuterol-budesonide vs albuterol was noted from as early as week 1, and patients receiving albuterol-budesonide qid also had nominally greater odds of achieving a clinically meaningful improvement in ACQ-7 score compared with those receiving albuterol alone.

The consequence of increasing SABA prescriptions has been evaluated in multiple studies across geographic regions, and is associated with increased risk of severe exacerbations (independent of maintenance therapy).^3^, ^4^, ^5^^,^^15^^,^^16^

The contribution of budesonide to the efficacy of albuterol-budesonide pMDI in reducing exacerbation risk has been previously reported in the phase 3 MANDALA trial, in which as-needed albuterol-budesonide 180/160 μg significantly reduced the risk of a severe asthma exacerbation in patients with moderate-to-severe asthma receiving a range of ICS-based maintenance regimens.^9^ The reduction in exacerbation risk observed in MANDALA is consistent with that reported with a free combination of beclomethasone in addition to SABA rescue in a recent real-world trial of Black and Latinx patients with uncontrolled moderate-to-severe asthma,^17^ and an earlier proof-of-concept study of as-needed use of albuterol-beclomethasone compared with as-needed albuterol alone in patients with mild asthma.^18^ In DENALI, severe exacerbations were assessed as an exploratory end point; fewer patients experienced severe exacerbation over the 12-week treatment period with albuterol-budesonide than with albuterol alone (2.0% and 2.5% for the 180/160 and 180/80 μg doses, respectively, vs 10.2%). Given DENALI was only a 12-week study, exacerbation data cannot be annualized and therefore should be interpreted with caution.

The scheduled qid dosing used in DENALI, although not reflective of the intended “real-world” as-needed use, provided the opportunity to evaluate the safety of regular use over 12 weeks of albuterol-budesonide at relatively high daily doses (compared with the mean 1.3 doses/d when used as needed in the MANDALA study^9^). Both doses of albuterol-budesonide were well tolerated, with an AE profile consistent with its monocomponents, and a low incidence of treatment-related AEs, serious AEs, or AEs leading to discontinuation. No new safety findings were observed in DENALI, and local ICS-associated AEs were low, despite the qid dosing employed in this trial. Consistent with the MANDALA trial,^9^ the most frequently reported AEs were nasopharyngitis and headache. The overall incidence and type of AEs were similar across treatment groups.

A strength of our trial was the randomized, double-blind design, with active comparators and a placebo arm (for assay sensitivity), and a hierarchical statistical testing procedure to control for multiplicity. The choice of active comparators (albuterol and budesonide) reflected the regulatory objective of the trial, that is, to demonstrate the contribution of each monocomponent to the efficacy of the combination. The inclusion of sites from three continents ensured the global generalizability of the findings. Despite a temporary halt to enrollment between March and May 2020 because of the global COVID-19 pandemic, target enrollment was met, few trial visits were missed, and there was also a low discontinuation rate.

The main limitation of the trial is the scheduled qid dosing which, although necessary to achieve the regulatory objectives of the trial, does not reflect the intended as-needed use. It is recognized, however, that many patients with poor asthma control who rely on their SABA rescue therapy may self-administer doses regularly at times of increasing symptoms or even in excess of this scheduled use. Other limitations include the lack of measurements of inflammatory markers; the small number of children and adolescents enrolled; and the permitted use of albuterol as needed for symptoms during the randomized treatment period, which may have resulted in an underestimation of the potential lung function benefit of albuterol-budesonide treatment. Finally, because of the inclusion of a placebo (plus as-needed albuterol) arm, DENALI enrolled patients with mild-to-moderate asthma receiving only SABA alone or low-dose ICS maintenance therapy plus as-needed SABA. The efficacy and safety of albuterol-budesonide pMDI as needed in patients with moderate-to-severe asthma receiving ICS-containing maintenance therapy has been previously reported.^14^

## Interpretation

This phase 3 trial in patients with mild-to-moderate asthma confirms the contribution of both albuterol and budesonide components to the lung function efficacy of albuterol-budesonide (thus satisfying the FDA combination rule), and its well-tolerated AE profile even at regular relatively high daily doses.

## Funding/Support

Bond Avillion 2 Development LP funded the trial and had a role in trial design, data collection, data analysis, data interpretation, and writing of the report. The corresponding author had full access to all the data and had final responsibility to submit for publication. Medical writing support was funded by AstraZeneca.

## Financial/Nonfinancial Disclosures

The authors have reported to *CHEST* the following: B. E. C. is an advisor for, has received consultancy fees from, and is on the speakers’ bureau for AstraZeneca, Boehringer Ingelheim, Genentech, GlaxoSmithKline, Novartis, Regeneron, and Sanofi Genzyme. E. I. reports personal fees from AB Science, Allergy and Asthma Network, Biometry, Equillium, Merck, NHLBI, 4D Pharma, Pneuma Respiratory, PPS Health, Regeneron, Sanofi Genzyme, and Sienna Biopharmaceutical; grants and personal fees from Amgen, AstraZeneca, Avillion, and Novartis; personal fees and nonfinancial support from Genentech, GlaxoSmithKline, and Teva; grants from Gossamer Bio; grants and nonfinancial support from Circassia; nonfinancial support from Boehringer Ingelheim; and other from Vorso Corp, outside the submitted work. R. B. reports receiving grants from AstraZeneca, Cure Kids, the Health Research Council of New Zealand, Genentech, and GlaxoSmithKline; receiving personal fees from AstraZeneca, Avillion, Cipla, and Theravance; serving on advisory boards for AstraZeneca, Avillion, and Theravance; and serving as the chair of the adolescent and adult asthma guidelines group for the Asthma and Respiratory Foundation NZ outside the submitted work. R. A. P. reports grants and advisory board support from AstraZeneca, Sanofi, Genentech, MedImmune, Origa Pharma, Amgen, RIFM, NIH, Regeneron, and Novartis. F. C. A., R. R., and A. D. are employees of Avillion. C. C., L. D., and E. J. are employees of, and own stock in, AstraZeneca. A. P. reports grants from Chiesi, AstraZeneca, GlaxoSmithKline, Boehringer Ingelheim, Teva, and Sanofi, and consulting fees, honoraria for lectures or advisory boards from Chiesi, AstraZeneca, GlaxoSmithKline, Boehringer Ingelheim, Novartis, Sanofi, IQVIA, Avillion, Elpen Pharmaceuticals, Menarini, Zambon, and Mundipharma.

## Supplementary Data

e-Online Data
