# Supplementary material for: Albuterol-Budesonide Pressurized Metered Dose Inhaler in Patients With Mild-to-Moderate Asthma: Results of the DENALI Double-Blind Randomized Controlled Trial
Source: Chest. 2023 Mar 30;164(3):585–95. doi: 10.1016/j.chest.2023.03.035 (PMC11898224; doi:10.1016/j.chest.2023.03.035)
Supplement: e-Online Data [file mmc1.docx]

**Supplementary Material**

Contents

[Assessment of possible impact of COVID-19 2](#_Toc134626133)

[Sample size re-estimations 2](#_Toc134626134)

[Supplementary Tables 3](#_Toc134626135)

[e-Table 1. Baseline demographic and clinical characteristics (full analysis set, all ages) 3](#_Toc134626136)

[e-Table 2: Adherence to randomized trial medication (full analysis set, ≥12 years) 4](#_Toc134626137)

[e-Table 3. Analysis of dual primary efficacy endpoints for all comparisons (efficacy population, ≥12 years) 5](#_Toc134626138)

[e-Table 4: Number of patients at each timepoint for evaluation of FEV_1_ AUC_0–6h_ least-squares mean change from baseline over 12 weeks 6](#_Toc134626139)

[e-Table 5. Tipping point analysis of the dual-primary efficacy endpoints (efficacy population, ≥12 years) 7](#_Toc134626140)

[e-Table 6. Reference-based imputation analysis (attributable estimand) of the dual-primary efficacy endpoints (efficacy population, ≥12 years) 8](#_Toc134626141)

[e-Table 7. Analysis of the dual-primary efficacy endpoints (efficacy population, including patients of all ages) 9](#_Toc134626142)

[e-Table 8. Change from baseline in trough FEV_1_ at Week 1 (efficacy population, ≥12 years) 10](#_Toc134626143)

[e-Table 9. Severe exacerbations (efficacy population, ≥12 years) 11](#_Toc134626144)

[e-Table 10. As-needed rescue therapy use (efficacy population, ≥12 years) 12](#_Toc134626145)

[e-Table 11. Local ICS-associated adverse events (safety population, all ages) 13](#_Toc134626146)

[Supplementary Figures 14](#_Toc134626147)

[e-Figure 1. Trial design, treatment visits and assessments 14](#_Toc134626148)

[e-Figure 2. Patients with an FEV_1_ response on Day 1 15](#_Toc134626149)

# Assessment of possible impact of COVID-19

Visits that were missed, delayed or completed remotely, due to COVID-19 were summarized descriptively. Patients unable to continue with procedures and scheduled assessments due to COVID-19 were withdrawn from the trial. Missing data because of COVID-19-related premature withdrawals or missed spirometry assessments or because of ACQ-7 data collected remotely because of COVID-19, were considered missing at random.

# Sample size re-estimations

Due to uncertainty around the variability from the supporting lung function data, two blinded sample size re-estimations were conducted after 44% and 65% of patients had completed 12 weeks’ treatment. At the first re-estimation, a standard deviation of 290 mL was observed for the blinded trough FEV_1_ data at Week 12, and sample size was increased to 1000 patients, as this was estimated to provide 90% power to detect a 100 mL difference in the change from baseline in trough FEV_1_ at Week 12. At the second re-estimation, a standard deviation of 320 mL was observed for the blinded trough FEV_1_ data at Week 12, providing 85% power to detect a 100 mL difference; this was considered sufficient, and the sample size was not increased further. Although variability was slightly increased at the second re‑estimation from the first re-estimation, the drop out rate was lower than originally estimated. As ≥80% power was maintained, the sample size was not further increased.

# Supplementary Tables

## e-Table 1. Baseline demographic and clinical characteristics (full analysis set, all ages)

|  | Albuterol–budesonide 180/160 μg  (N=197) | Albuterol–budesonide 180/80 μg  (N=204) | Albuterol 180 μg  (N=200) | Budesonide 160 μg  (N=199) | Placebo  (N=199) |
| --- | --- | --- | --- | --- | --- |
| **Age** |  |  |  |  |  |
| **Mean (SD), years** | 50.0 (15.8) | 48.7 (16.8) | 47.0 (16.8) | 48.3 (15.8) | 48.6 (15.8) |
| **Distribution, n (%)** |  |  |  |  |  |
| **≥4 to <12** | 0 | 3 (1.5) | 4 (2.0) | 0 | 3 (1.5) |
| **≥12 to <18** | 4 (2.0) | 7 (3.4) | 5 (2.5) | 5 (2.5) | 4 (2.0) |
| **≥18 to <65** | 154 (78.2) | 155 (76.0) | 158 (79.0) | 161 (80.9) | 161 (80.9) |
| **≥65** | 39 (19.8) | 39 (19.1) | 33 (16.5) | 33 (16.6) | 31 (15.6) |
| **Female sex, n (%)** | 125 (63.5) | 128 (62.7) | 120 (60.0) | 120 (60.3) | 127 (63.8) |
| **Race, n (%)** |  |  |  |  |  |
| **White** | 179 (90.9) | 185 (90.7) | 166 (83.0) | 180 (90.5) | 174 (87.4) |
| **Black or African American** | 14 (7.1) | 15 (7.4) | 30 (15.0) | 18 (9.0) | 19 (9.5) |
| **Asian** | 1 (0.5) | 0 | 0 | 0 | 1 (0.5) |
| **American Indian or Alaska Native** | 1 (0.5) | 0 | 1 (0.5) | 0 | 1 (0.5) |
| **Other** | 2 (1.0) | 4 (2.0) | 3 (1.5) | 1 (0.5) | 4 (2.0) |
| **Pre-bronchodilator FEV_1_, mean (SD)** |  |  |  |  |  |
| **Volume, L** | 2.1 (0.6) | 2.1 (0.7) | 2.2 (0.7) | 2.1 (0.7) | 2.1 (0.7) |
| **% predicted normal** | 68.8 (13.1) | 70.3 (14.8) | 69.9 (13.1) | 68.9 (13.8) | 68.7 (14.8) |
| **Reversibility in FEV_1_ , mean (SD), %^a^** | 28.2 (13.5) | 30.1 (12.8) | 27.4 (13.4) | 28.7 (14.4) | 28.5 (14.3) |
| **Pre-trial background ICS therapy, n (%)** | 93 (47.2) | 99 (48.5) | 94 (47.0) | 95 (47.7) | 95 (47.7) |
| **ACQ-7 overall score, mean (SD)** | 2.2 (0.7) | 2.2 (0.6) | 2.2 (0.7) | 2.1 (0.6) | 2.2 (0.7) |

ACQ-7, Asthma Control Questionnaire (7 item); FEV_1_, forced expiratory volume in 1 second; SD, standard deviation.

N=999; one patient did not receive any trial medication and one duplicate patient was also excluded from the full analysis set.

^a^Reversibility was tested during screening and calculated as follows: (post-bronchodilator FEV_1_ (L) − pre-bronchodilator FEV_1_ (L)) / pre-bronchodilator FEV_1_ (L) x 100. Screening was defined as the time before the first administration of sponsor-provided albuterol during the run-in period.

## e-Table 2: Adherence to randomized trial medication (full analysis set, ≥12 years)

|  | | **Albuterol–budesonide 180/160 µg (N=197)** | **Albuterol–budesonide 180/80 µg (N=201)** | **Albuterol 180 µg  (N=196)** | **Budesonide 160 µg (N=199)** | **Placebo (N=196)** |
| --- | --- | --- | --- | --- | --- | --- |
| **Overall adherence (%)** | Mean (SD) | 89.7 (11.0) | 89.3 (13.6) | 89.5 (13.0) | 89.5 (10.9) | 89.2 (12.4) |
|  | Median | 93.8 | 93.8 | 95.0 | 92.8 | 93.2 |
| **Patients with ≥ 80% adherence** | n (%) | 169 (85.8) | 170 (84.6) | 163 (83.2) | 162 (81.4) | 166 (84.7) |

Treatment adherence was calculated from eDiary records as the number of inhalations of trial medication taken as a proportion of the expected 8 inhalations/day of trial medication during the randomized treatment period.

SD, standard deviation

## e-Table 3. Analysis of dual primary efficacy endpoints for all comparisons (efficacy population, ≥12 years)

| **Primary efficacy endpoint** | **Comparisons** | **LSM** | **Comparison between groups** | |
| --- | --- | --- | --- | --- |
|  |  |  | **Difference in LSM (95% CI)** | ***P* value**  **(2-sided)** |
| **FEV_1_ AUC_0-6h_, change from baseline over 12 weeks (mL)^a^** | Albuterol 180 µg (n=195) vs. placebo (n=196) | 157.2 vs. 96.7 | 60.5 (7.7, 113.4) | .025 |
|  | Budesonide 160 µg (n=199) vs. placebo (n=196) | 178.0 vs. 96.7 | 81.3 (28.8, 133.7) | .002 |
|  | Albuterol–budesonide 180/80 μg (n=200) vs. placebo (n=196) | 242.2 vs. 96.7 | 145.5 (93.0, 197.9) | <.001 |
|  | Albuterol–budesonide 180/160 μg (n=197) vs. placebo (n=196) | 258.6 vs. 96.7 | 161.9 (109.4, 214.5) | <.001 |
|  | Albuterol–budesonide 180/80 μg (n=200) vs. albuterol 180 μg (n=195) | 242.2 vs. 157.2 | 84.9 (32.3, 137.5) | .002 |
|  | Albuterol–budesonide 180/160 μg (n=197) vs. albuterol 180 μg (n=195) | 258.6 vs. 157.2 | 101.4 (48.8, 154.1) | <.001 |
|  | Albuterol–budesonide 180/80 μg (n=200) vs. budesonide 160 μg (n=199) | 242.2 vs. 178.0 | 64.2 (12.1, 116.4) | .016 |
|  | Albuterol–budesonide 180/160 μg (n=197) vs. budesonide 160 μg (n=199) | 258.6 vs. 178.0 | 80.7 (28.4, 132.9) | .003 |
|  | Albuterol–budesonide 180/160 μg (n=197) vs. albuterol–budesonide 180/80 μg (n=200) | 258.6 vs. 242.2 | 16.5 (–35.8, 68.8) | .536 |
| **Trough FEV_1_, change from baseline at Week 12 (mL)** | Albuterol 180 μg (n=172) vs. placebo (n=175) | 2.7 vs. 35.6 | –32.9 (–102.8, 36.9) | .355 |
|  | Budesonide 160 μg (n=187) vs. placebo (n=175) | 108.9 vs. 35.6 | 73.3 (4.4, 142.2) | .037 |
|  | Albuterol–budesonide 180/80 μg (n=184) vs. placebo (n=175) | 123.5 vs. 35.6 | 87.9 (18.8, 156.9) | .013 |
|  | Albuterol–budesonide 180/160 μg (n=186) vs. placebo (n=175) | 135.5 vs. 35.6 | 99.9 (30.9, 168.8) | .005 |
|  | Albuterol–budesonide 180/80 μg (n=184) vs. albuterol 180 μg (n=172) | 123.5 vs. 2.7 | 120.8 (51.5, 190.1) | <.001 |
|  | Albuterol–budesonide 180/160 μg (n=186) vs. albuterol 180 μg (n=172) | 135.5 vs. 2.7 | 132.8 (63.6, 201.9) | <.001 |
|  | Albuterol–budesonide 180/80 μg (n=184) vs. budesonide 160 μg (n=187) | 123.5 vs. 108.9 | 14.6 (–53.6, 82.8) | .675 |
|  | Albuterol–budesonide 180/160 μg (n=186) vs. budesonide 160 μg (n=187) | 135.5 vs. 108.9 | 26.6 (–41.6, 94.7) | .444 |
|  | Albuterol–budesonide 180/160 μg (n=186) vs. albuterol–budesonide 180/80 (n=184) | 135.5 vs. 123.5 | 12.0 (–56.3, 80.3) | .731 |

AUC_0-6h_, area under the curve from 0 to 6 hours post dose; CI, confidence interval; FEV_1_, forced expiratory volume in 1 second; LSM, least squares mean.

^a^The LSM was calculated as the difference between treatment groups average over all time points during the treatment period.

## e-Table 4: Number of patients at each timepoint for evaluation of FEV_1_ AUC_0–6h_ least-squares mean change from baseline over 12 weeks

| **Treatment** | **Number of patients** | | | | | |
| --- | --- | --- | --- | --- | --- | --- |
|  | **Day 1** | **Week 1** | **Week 4** | **Week 8** | **Week 12** | **Average** |
| **Albuterol–budesonide 180/160 µg** | 197 | 188 | 184 | 185 | 187 | 197 |
| **Albuterol–budesonide 180/80 µg** | 200 | 193 | 183 | 182 | 184 | 200 |
| **Albuterol 180 µg** | 195 | 185 | 177 | 174 | 173 | 195 |
| **Budesonide 160 µg** | 199 | 190 | 186 | 187 | 187 | 199 |
| **Placebo** | 196 | 191 | 180 | 173 | 175 | 196 |

AUC_0-6h_, area under the curve from 0 to 6 hours post dose; FEV_1_, forced expiratory volume in 1 second.

## e-Table 5. Tipping point analysis of the dual-primary efficacy endpoints (efficacy population, ≥12 years)

| **Primary efficacy endpoint** | **Penalty (mL)** | **Comparisons** | **LSM^a^** | **Comparison between groups** | |
| --- | --- | --- | --- | --- | --- |
|  |  |  |  | **Difference in LSM (95% CI)** | ***P* value**  **(2-sided)** |
| FEV_1_ AUC_0-6h_, change from baseline over 12 weeks (mL)^a^ | 122 | Albuterol 180 µg (n=196) vs. placebo (n=196) | 151.8 vs. 97.4 | 54.4 (1.8, 107.0) | .043 |
|  |  | Albuterol–budesonide 180/160 µg (n=197) vs. placebo (n=196) | 258.5 vs. 97.4 | 161.2 (108.8, 213.6) | <.001 |
|  |  | Albuterol–budesonide 180/160 µg (n=197) vs. budesonide 160 µg (n=199) | 258.5 vs. 178.3 | 80.2 (28.1, 132.3) | .003 |
| Trough FEV_1_, change from baseline at Week 12 (mL) | 66 | Budesonide 160 µg (n=199) vs. placebo (n=196) | 107.1 vs. 34.6 | 72.5 (2.5, 142.5) | .042 |
|  |  | Albuterol–budesonide 180/160 µg (n=197) vs. placebo (n=196) | 132.6 vs. 34.6 | 98.0 (28.2, 167.8) | .006 |
|  |  | Albuterol–budesonide 180/160 µg (n=197) vs. albuterol 180 µg (n=196) | 132.6 vs. –0.2 | 132.8 (64.3, 201.4) | <.001 |
|  |  | Albuterol–budesonide 180/80 µg (n=201) vs. placebo (n=196) | 121.0 vs. 34.6 | 86.4 (16.8, 156.0) | .015 |
|  |  | Albuterol–budesonide 180/80 µg (n=201) vs. albuterol 180 µg (n=196) | 121.0 vs. –0.2 | 121.3 (52.3, 190.2) | <.001 |

AUC_0-6h_, area under the curve from 0 to 6 hours post dose; CI, confidence interval; FEV_1_, forced expiratory volume in 1 second; LSM, least squares mean.

^a^The LSM was calculated as the difference between treatment groups average over all time points during the treatment period.

The responses for post-treatment discontinuation data in the comparator arms are imputed under a missing not at random (MNAR) assumption, in which a penalty is applied. All other data are imputed under a missing at random assumption. The tipping point analysis incrementally penalized the missing data under the MNAR assumption until a non-statistically significant comparison was observed in the sequential testing strategy. The maximum pre-specified penalties of 122.0 mL for change from baseline in FEV_1_ AUC_0-6h_ and 66.0 mL for change from baseline in trough FEV_1_are reported here.

## e-Table 6. Reference-based imputation analysis (attributable estimand) of the dual-primary efficacy endpoints (efficacy population, ≥12 years)

| **Primary efficacy endpoint** | **Comparisons** | **LSM** | **Comparison between groups** | |
| --- | --- | --- | --- | --- |
|  |  |  | **Difference in LSM (95% CI)** | ***P* value**  **(2-sided)** |
| **FEV_1_ AUC_0-6h_, change from baseline over  12 weeks (mL)^a^** | Albuterol 180 µg (n=196) vs. placebo (n=196) | 144.4 vs. 89.0 | 55.4 (2.7, 108.1) | .039 |
|  | Albuterol–budesonide 180/160 µg (n=197) vs. placebo (n=196) | 256.9 vs. 89.0 | 168.0 (115.4, 220.5) | <.001 |
|  | Albuterol–budesonide 180/160 µg (n=197) vs. budesonide 160 µg (n=199) | 257.7 vs. 177.1 | 80.6 (28.5, 132.7) | .002 |
| **Trough FEV_1_, change from baseline at  Week 12 (mL)** | Budesonide 160 µg (n=199) vs. placebo (n=196) | 104.9 vs. 18.1 | 86.8 (17.4, 156.2) | .014 |
|  | Albuterol–budesonide 180/160 µg (n=197) vs. placebo (n=196) | 129.2 vs. 18.1 | 111.1 (41.8, 180.4) | .002 |
|  | Albuterol–budesonide 180/160 µg (n=197) vs. albuterol 180 µg (n=196) | 128.6 vs. –21.7 | 150.3 (81.3, 219.3) | <.001 |
|  | Albuterol–budesonide 180/80 µg (n=201) vs. placebo (n=196) | 118.6 vs. 18.1 | 100.4 (31.1, 169.8) | .005 |
|  | Albuterol–budesonide 180/80 µg (n=201) vs. albuterol 180 µg (n=196) | 118.2 vs. –21.7 | 139.9 (70.5, 209.2) | <.001 |

AUC_0-6h_, area under the curve from 0 to 6 hours post dose; CI, confidence interval; FEV_1_, forced expiratory volume in 1 second; LSM, least squares mean.

^a^The LSM was calculated as the difference between treatment groups average over all time points during the treatment period.

Missing data and post-treatment discontinuation data are imputed using multiple imputation of 50 imputed datasets. Post-treatment discontinuation data due to tolerability or lack of efficacy are imputed based on the 5^th^ percentile of the corresponding reference group for each treatment comparison made. All other missing data are imputed under a missing at random assumption.

## e-Table 7. Analysis of the dual-primary efficacy endpoints (efficacy population, including patients of all ages)

| **Primary efficacy endpoint** | **Step in testing sequence** | **Comparisons** | **LSM** | **Comparison between groups** | |
| --- | --- | --- | --- | --- | --- |
|  |  |  |  | **Difference in LSM (95% CI)** | ***P* value**  **(2-sided)** |
| **FEV_1_ AUC_0-6h_, change from baseline over 12 weeks (mL)^a^** | 1 | Albuterol 180 μg (n=199) vs. placebo (n=199) | 159.2 vs. 94.0 | 65.2 (12.7, 117.7) | .015 |
|  | 2 | Albuterol–budesonide 180/160 μg (n=197) vs. placebo (n=199) | 260.1 vs. 94.0 | 166.1 (113.6, 218.6) | <.001 |
|  | 3 | Albuterol–budesonide 180/160 μg (n=197) vs. budesonide 160 μg (n=199) | 260.1 vs. 179.8 | 80.3 (28.0, 132.7) | .003 |
| **Trough FEV_1_, change from baseline at Week 12 (mL)** | 4 | Budesonide 160 μg (n=187) vs. placebo (n=178) | 111.3 vs. 32.7 | 78.5 (9.7, 147.4) | .025 |
|  | 5 | Albuterol–budesonide 180/160 μg (n=186) vs. placebo (n=178) | 137.3 vs. 32.7 | 104.5 (35.6, 173.4) | .003 |
|  | 6 | Albuterol–budesonide 180/160 μg (n=186) vs. albuterol 180 μg (n=176) | 137.3 vs. 12.3 | 124.9 (55.9, 193.9) | <.001 |
|  | 7 | Albuterol–budesonide 180/80 μg (n=187) vs. placebo (n=178) | 121.3 vs. 32.7 | 88.5 (19.8, 157.3) | .012 |
|  | 8 | Albuterol–budesonide 180/80 μg (n=187) vs. albuterol 180 μg (n=176) | 121.3 vs. 12.3 | 108.9 (40.0, 177.8) | .002 |

AUC_0-6h_, area under the curve from 0 to 6 hours post dose; CI, confidence interval; FEV_1_, forced expiratory volume in 1 second; LSM, least squares mean.

^a^The LSM was calculated as the difference between treatment groups average over all time points during the treatment period.

## e-Table 8. Change from baseline in trough FEV_1_ at Week 1 (efficacy population, ≥12 years)

| **Comparisons** | **LSM** | **Comparison between groups** | |
| --- | --- | --- | --- |
|  |  | **Difference in LSM (95% CI)** | ***P* value**  **(2-sided)** |
| Albuterol 180 μg (n=185) vs. placebo (n=191) | -0.8 vs. 41.3 | -42.1 (-101.9, 17.8) | .170 |
| Budesonide 160 μg (n=190) vs. placebo (n=191) | 93.4 vs. 41.3 | 52.1 (-7.4, 111.5) | .086 |
| Albuterol–budesonide 180/80 μg (n=192) vs. placebo (n=191) | 72.0 vs. 41.3 | 30.7 (-28.6, 90.1) | .310 |
| Albuterol–budesonide 180/160 μg (n=188) vs. placebo (n=191) | 107.2 vs. 41.3 | 65.9 (6.3, 125.4) | .030 |
| Albuterol–budesonide 180/80 μg (n=192) vs. albuterol 180 μg (n=185) | 72.0 vs. -0.8 | 72.8 (13.1, 132.5) | .017 |
| Albuterol–budesonide 180/160 μg (n=188) vs. albuterol 180 μg (n=185) | 107.2 vs. -0.8 | 107.9 (48.1, 167.8) | <.001 |
| Albuterol–budesonide 180/80 μg (n=192) vs. budesonide 160 μg (n=190) | 72.0 vs. 93.4 | -21.3 (-80.5, 37.9) | .480 |
| Albuterol–budesonide 180/160 μg (n=188) vs. budesonide 160 μg (n=190) | 107.2 vs. 93.4 | 13.8 (-45.6, 73.2) | .648 |

CI, confidence interval; LSM, least squares mean.

## e-Table 9. Severe exacerbations (efficacy population, ≥12 years)

| Severe exacerbations^a^ | Albuterol–budesonide  180/160 µg (N=197) | Albuterol–budesonide  180/80 µg (N=201) | Albuterol 180 µg (N=196) | Budesonide  160 µg (N=199) | Placebo (N=196) |
| --- | --- | --- | --- | --- | --- |
| **Patients with ≥1 severe exacerbation, n (%)** | 4 (2.0) | 5 (2.5) | 20 (10.2) | 4 (2.0) | 14 (7.1) |
| **Days with severe exacerbations**  **Total days**  **Per patient, days, mean (SD)** | 22  0.1 (0.9) | 31  0.2 (1.0) | 130  0.7 (2.1) | 42  0.2 (1.5) | 85  0.4 (2.1) |

^a^Defined as ≥1 of the following: a ≥3-day course of oral corticosteroids or corresponding single injectable dose, an emergency room or urgent care visit due to asthma that required systemic corticosteroids, or hospitalization due to asthma.

SD, standard deviation.

## e-Table 10. As-needed rescue therapy use (efficacy population, ≥12 years)

| **No. of inhalations/day of as-needed rescue therapy** | **Albuterol–budesonide 180/160 µg (N=197)** | **Albuterol–budesonide 180/80 µg (N=201)** | **Albuterol 180 µg (N=196)** | **Budesonide 160 µg (N=199)** | **Placebo (N=196)** |
| --- | --- | --- | --- | --- | --- |
| **Treatment average, mean (SD)** | 1.27 (1.39) | 1.43 (1.66) | 1.92 (2.0) | 1.40 (1.60) | 1.94 (1.95) |
| **Change from baseline, LSM (SE)** | –1.48 (0.11)** | –1.36 (0.11)* | –0.95 (0.11) | –1.29 (0.11) | –0.73 (0.11) |

Change from baseline was evaluated using an analysis of covariance.
*p<0.01 vs albuterol; **p<0.001 vs albuterol

LSM, least squares mean; SD, standard deviation; SE, standard error.

## e-Table 11. Local ICS-associated adverse events (safety population, all ages)

| **n (%)** | **Albuterol–budesonide 180/160 μg  (N=197)** | **Albuterol–budesonide 180/80 μg  (N=204)** | **Albuterol  180 μg  (N=201)** | **Budesonide  160 μg  (N=199)** | **Placebo (N=199)** |
| --- | --- | --- | --- | --- | --- |
| **Oral candidiasis** | 1 (0.5) | 1 (0.5) | 0 | 0 | 0 |
| **Oropharyngeal candidiasis** | 2 (1.0) | 0 | 0 | 0 | 0 |
| **Dysphonia** | 4 (2.0) | 1 (0.5) | 0 | 2 (1.0) | 0 |

Preferred terms from the Medical Dictionary for Regulatory Activities (MedDRA) version 24.0 were used. n=number of patients. Patients with multiple events in the same preferred term were counted only once in that preferred term.

# Supplementary Figures

## e-Figure 1. Trial design, treatment visits and assessments


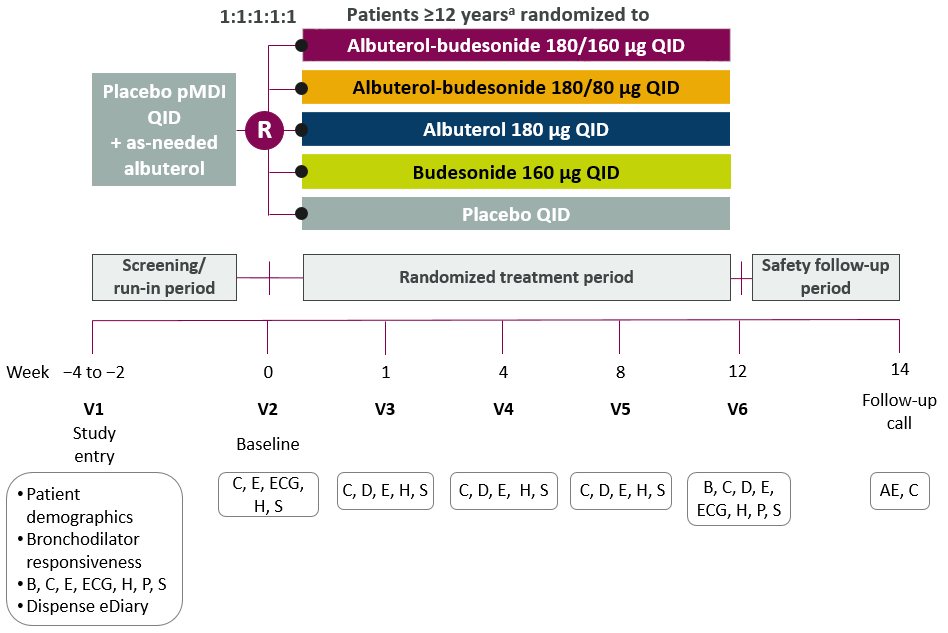
ACQ-7, Asthma Control Questionnaire; AE, adverse event; B, collect blood samples for clinical chemistry and hematology assessments; C, concomitant medications; D, eDiary review; E, efficacy assessment (FEV_1_, ACQ-7); ECG, electrocardiogram; FEV_1_, forced expiratory volume in 1 second; H, medical and surgical history; P, physical examination; pMDI, metered-dose inhaler; QID, four-times daily; S, safety assessment (AEs, vital signs, medical history, pregnancy test); V, visit.

Participants were recruited and enrolled at participating sites.

^a^Randomized treatment groups shown only for adolescents and adults (patients aged ≥12 years). Children (4–11 years) were randomized 1:1:1 to Albuterol–budesonide 180/80 μg QID, albuterol 180 μg QID, or placebo QID; *a priori*, children (n=10) were excluded from efficacy analyses.

## e-Figure 2. Patients with an FEV_1_ response on Day 1


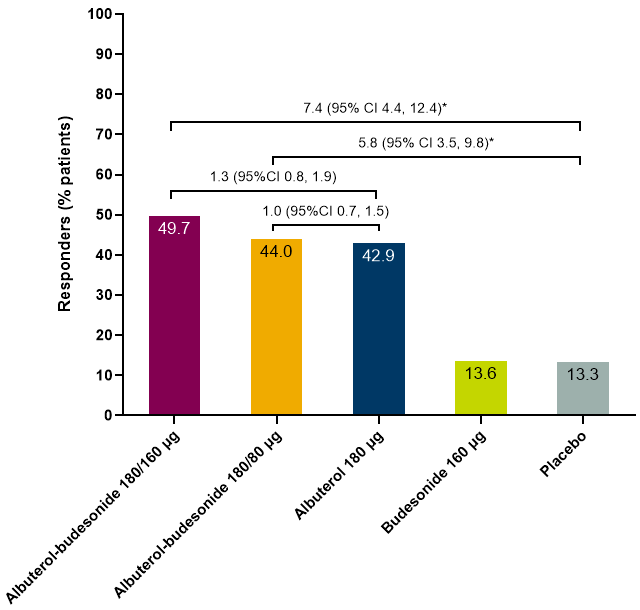


FEV_1_, forced expiratory volume in 1 second; CI, confidence interval.

**P* < .001 vs placebo**.**

A responder was defined as a subject with an observed increase from baseline FEV_1_ of ≥15% within 30 minutes post dose. Odds ratio (95% CI) albuterol vs placebo: 5.8 (3.5, 9.8); *P* < .001. Odds ratio (95% CI) budesonide vs placebo: 1.0 (0.6, 1.9); *P* = .9.
